# In developed countries male circumcision prevalence is inversely related to HIV prevalence

**DOI:** 10.1186/s13584-015-0034-7

**Published:** 2015-08-13

**Authors:** Brian J. Morris, Jeffrey D. Klausner

**Affiliations:** School of Medical Sciences and Bosch Institute, University of Sydney, Sydney, New South Wales Australia; Division of Infectious Diseases, Department of Medicine, David Geffen School of Medicine, University of California Los Angeles, California, USA

**Keywords:** Circumcision, Male, HIV, Rate, Europe, Israel, Public health, Policy

## Abstract

A study by Chemtob and co-workers found significantly lower prevalence of HIV amongst heterosexual men and women in Israel compared with the Netherlands and France. Risk factors for heterosexual HIV infection in these countries were similar, apart from one, namely, a strikingly higher prevalence of male circumcision (MC) in Israel compared with the Netherlands and France. It is now well established that MC protects heterosexual men against becoming infected with HIV during sexual intercourse with an infected woman. In epidemic settings, such as countries in sub-Saharan Africa, in which heterosexual contact is the primary driver for HIV infection, MC is being implemented to reduce HIV prevalence. The results of the new study by Chemtob and co-workers support the evidence and recent polices in the United States advocating MC to reduce the spread of HIV. While prevalence in developed countries is generally low, it is rising. In the long term, neonatal MC is the most desirable option, since not only is it simpler, safer, cheaper and more convenient than MC later, it provides immediate protection from infections, penile inflammation, genital cancers and physical problems. It is also cost-effective. European countries have not supported MC for its public health benefits. The new findings add to calls for European and other countries with low MC prevalence to consider developing evidence-based policies favoring MC in order to reduce HIV and other infections and diseases and at the same time reduce suffering, mortality and the cost of treating these.

## Male circumcision protects against HIV infection

The evidence that male circumcision (MC) can substantially reduce HIV infection in men during heterosexual intercourse is now well accepted, leading to its adoption as an HIV prevention strategy in high prevalence settings of sub-Saharan Africa [1]. The policy followed the publication of results from three randomized controlled trials (RCTs) in South Africa [2], Kenya [3] and Uganda [4]. Further support has been provided by meta-analyses [5–8], effectiveness studies in the implementation of MC [9], follow-up of RCT study participants in which protection reached 70 % [10–12], and biological evidence [13]. However, the relevance of MC for HIV prevention in low prevalence settings, as applies to developed nations, has been less clear.

## New findings for developed countries

A recent article in *Israel Journal of Health Policy Research* by Chemtob and colleagues has provided much-needed evidence demonstrating that MC was associated with reduced HIV acquisition in heterosexuals in countries in which HIV prevalence is low [14]. The study found the rate of newly diagnosed heterosexual HIV cases in Israel, where MC prevalence exceeds 90 %, was 0.46 (range 0.26–0.70) per 100,000 of the population per year over the period 2004–2010. The annual incidence of HIV infection in men in Israel was on average 6 times lower than in the Netherlands (mean 2.0 annual cases per 100,000 of the population; range 1.9–2.3) and France (mean 3.3; range 2.7–3.5) where the MC prevalence in both is less than 10 %. HIV prevalence was also lower in women in Israel (0.20; range 0.10–0.34), where the number of cases per year were 10 times fewer over that period when compared with the annual number of cases in women in the Netherlands (1.4; range 1.1–2.1) and France (2.6; range 2.4–3.1). The authors excluded cases originating in men who have sex with men and intravenous drug users, each of which represent high-risk groups. In addition, they also excluded cases of heterosexual HIV transmission originating from countries with generalized HIV epidemics. Migrants were, moreover, unlikely to contribute very much to HIV infections in the general heterosexual populations of each country.

While inter-country comparisons are subject to the influence of confounding factors, including sexual behavior, the three countries examined are fairly similar in just about every aspect of known risk factors for sexually acquired HIV infection, apart from MC prevalence. Those factors included the number of sex partners and condom use. The proportion of people tested for HIV in the past year was highest in Israel. The authors suggest that infection of women by a small proportion of men who engage in sexual intercourse with both women and other men was of very limited impact.

## HIV was once a rare virus

In those countries, mostly in sub-Saharan Africa, where HIV prevalence is at epidemic levels, HIV was once rare or nonexistent. Had MC been introduced as a preventive measure before the three trials were completed in 2007 then many infections would have been averted and lives could have been saved [15].

## HIV is rising in developed countries

In confirmation of the basis of the findings by Chemtob *et al*., the protective effect of MC against acquisition of HIV in heterosexual men applies just as well in another low prevalence country, the USA [16]. The proportion of HIV cases attributable to heterosexual contact has, moreover, risen substantially with time in developed countries [17, 18]. National statistics for Australia show that 25 % of cases involved heterosexual contact [18]. After excluding cases from a high prevalence country, the number of cases whose exposure to HIV was attributed to heterosexual contact has increased by 28 % over the past decade [18], 29 % of these being in individuals born in Australia [18, 19]. There has been a steady rise in HIV prevalence in the WHO European Region [20], notably in some non-drug injecting, heterosexual populations in Eastern Europe, as well as in Central Asia [21]. In African Americans in the USA, HIV rates are rising faster than almost all other groups in that country [22]. The US Centers for Disease Control and Prevention (CDC) has recommended MC for HIV prevention in high prevalence groups such as those [23]. Protection against HIV infection was an important component of recent MC policy recommendations by the US CDC [24] and the American Academy of Pediatrics [25].

## The looming treatment burden

A recent study in the Netherlands highlighted the looming medical burden as a result of an anticipated enormous increase in multiple morbidities and drug interactions in aging HIV-infected patients on combination antiretroviral therapy [26].

## Neonatal circumcision preferable

In the long term, neonatal MC appears to offer advantages over adult MC for prophylaxis against HIV and other infectious diseases globally [27]. That is because neonatal MC is cheaper, simpler, safer, more convenient, averts concerns about premature resumption of sex during wound healing and provides a risk-benefit ratio of 100:1 in favor [28], as noted by the US CDC [24]. Adverse events are uncommon, virtually all being minor, easy to treat and with complete resolution [25, 28, 29].

## Effectiveness against HIV

The 60 % or higher efficacy of MC in protecting heterosexual men against HIV infection [5, 9–12] makes MC more effective than condoms. That is because, even though condoms are 80 % protective against HIV infection *if used consistently and correctly* [30, 31], a Cochrane systematic review of RCTs of condom use found, “little clinical evidence of effectiveness” and no “favorable results” for HIV prevention [32]. Unlike condoms, MC is a one-off procedure that does not require an item to be applied or administered each time a man has sexual intercourse. Nevertheless both MC and condom use should be advocated.

By reducing HIV prevalence in heterosexual men, MC will help reduce HIV prevalence in women [33] and children [34]. It will also help lower risks for other sexually transmitted infections [28, 35–58], including those that exacerbate HIV risk [41–44].

Finally, MC has been shown to be the most cost-effective of all the available interventions for HIV prevention [59]. Calculations for the US have shown that if MC prevalence were to fall from the current high levels of 80 to 10 %, as typically seen in Europe, direct costs for treatment of urinary tract and sexually transmitted infections, including HIV, would increase by US$4.4 billion for 10 annual birth cohorts [60].

## Conclusion

The new findings by Chemtob *et al*. have broad implications for efforts to arrest the continued spread of HIV in the heterosexual community of developed countries in which HIV prevalence is currently low. While routine MC will be easier in countries such as Israel and the US that already enjoy a cultural or religious tradition of infant MC, it presents a challenge in countries such as those in Europe in which a cultural bias against MC exists amongst the majority [61].

## Commentary on

Chemtob D, Op de Coul E, Van Sighem A, Mor Z, Cazein F, et al. Impact of male circumcision among heterosexual HIV cases: comparison between three low prevalence countries. Israel J Health Policy. 2015;4:36.

## Abbreviations

MC: Male circumcision
HIV: Human immunodeficiency virus
CDC: Centers for Disease Control and Prevention
US: United States of America
WHO: World Health Organization
